# Supplementary material for: Effectiveness of virtual reality–based exercise rehabilitation for enhancing functional outcomes and quality of life in adult cancer survivors: A systematic review of randomized controlled trials
Source: J Cancer Surviv. 2025 Nov 26;20(5):2760–71. doi: 10.1007/s11764-025-01923-0 (PMC13723611; doi:10.1007/s11764-025-01923-0)
Supplement: Supplementary file 1 [file 11764_2025_1923_MOESM1_ESM.docx]

Effectiveness of Virtual Reality–Based Exercise Rehabilitation for Enhancing Functional Outcomes and Quality of Life in Adult Cancer Survivors: A Systematic Review of Randomized Controlled Trials

Manar Eid^1^, Haytham Alkerdasy^2^, Amina Hilmy^2^, Abdelrahman Sameh^2^, Amal Ashraf^2^

1: 5^th^ year student at faculty of physical therapy, Benha University

2: intern student at faculty of physical therapy, Benha University

Corresponding author details:

Manar Eid

[Manareid126@gmail.com](mailto:Manareid126@gmail.com)

01554510545

**Abstract:**

**Purpose:** This systematic review aims to synthesize randomized controlled trials (RCTs) evaluating the effectiveness and safety of virtual reality–based exercise rehabilitation (VRER) in adult cancer survivors

**Methods:** Following PRISMA 2020, PubMed, Scopus, Web of Science, and Cochrane Library were searched up to November 2024. Only RCTs of VR-based exercise in adult patients with cancer were included. Risk of bias was assessed using the Cochrane ROB2 tool. Outcomes were narratively synthesized across strength/range of motion, functional status, quality of life, physical performance, and adverse events.

**Results:** Eight RCTs involving 359 participants with breast, prostate, brain, or combined cancer types were included. VRER increased shoulder mobility and reduced fear of movement compared with usual care. Functional status, specifically activities of daily living, showed consistent improvement. Physical performance improved including better balance, endurance, and activity level. Three trials reported quality-of-life advantages, mainly in physical and vitality domains, though heterogeneity of measures limited comparison. Safety was good with only mild, transient symptoms and no serious adverse events.

**Conclusions**: VRER has promising effects on mobility, daily function, physical performance, and quality of life in cancer survivors, with strong adherence and safety.

**Implications for Cancer Survivors:** VR-based exercise is an effective, safe adjunct to conventional rehabilitation. By improving mobility, adherence, and well-being, it offers benefits and can be integrated into survivorship care.

**Keywords:** Virtual reality; Cancer rehabilitation; Exercise therapy; Quality of life; Survivorship

**Introduction**

Cancer remains a significant worldwide health challenge, and 19.3 million new diagnoses and 10 million cancer-related deaths were estimated in 2020 [1]. Early diagnosis, multimodal treatment, and supportive care have significantly contributed to rising survival rates, as well as rising numbers of cancer survivors worldwide [2]. With this demographic shift, more instances of chronic cancer-related dysfunctions (CRDs), such as physical, cognitive, and psychosocial impairments, have been observed. These include cancer-related fatigue (CRF), musculoskeletal dysfunction, chemotherapy-induced peripheral neuropathy (CIPN), cognitive impairment, and pain syndromes, all of which significantly impact quality of life (QoL) and functional independence [3].

Conventional rehabilitation interventions, such as structured exercise therapy, physical therapy, and cognitive-behavioral therapy, are recommended for managing CRDs in oncology [4]. Evidence indicates that these approaches can enhance physical function, reduce fatigue, and improve psychological resilience, thereby supporting cancer survivors in regaining independence and maintaining daily activities [5,6]. Despite their proven benefits, the clinical impact of conventional rehabilitation strategies is often constrained by barriers such as geographic remoteness, high treatment costs, limited availability of specialized services, mobility restrictions, and low patient adherence [7,8]. These limitations highlight the urgent need for innovative, accessible, and engaging rehabilitation approaches that can overcome participation barriers while supporting sustained engagement.

Given these challenges, there is a growing interest in technology-driven rehabilitation solutions, particularly virtual reality–based exercise rehabilitation (VRER), which offers the potential to address these shortcomings. Virtual reality–based exercise rehabilitation (VRER) is an innovative technology-based intervention, integrating physical exercise with interactive immersive virtual environments [9]. Immersive virtual systems which are typically delivered via head-mounted displays (HMDs) generate three-dimensional lifelike representations that allow patients to engage in precise motor and cognitive training in a secure and controlled environment [10]. Non-immersive VR systems like screen-based exergaming systems offer gamified exercise programs that promote movement, balance, and coordination as well as real-time visual and auditory feedback [11-13]. Both modalities can be individualized to meet the needs of patients, enhance motivation, and give precise monitoring of improvement.

In addition to physical advantages, VRER also has psychological and cognitive benefits, including anxiety and depression reduction and improvement in executive function and working memory [14-15]. The gamification factor helps reduce psychological barriers, such as fear of movement and improve self-efficacy, which are prevalent in cancer survivors [14,15]. Moreover, VRER can be integrated into telerehabilitation frameworks, where supervised, home-based exercise participation is possible and suitable for immunocompromised subjects and travel-limited patients [16]. Even with heightened clinical interest, current evidence on VRER in cancer rehabilitation is heterogeneous and methodologically varied with considerable variation in patient groups, intervention protocols, type of VR system, and outcomes measures [17]. Previous reviews have included non-randomized and quasi-experimental study designs limiting the strength and generalizability of their findings [18].

To overcome these limitations, this systematic review focuses exclusively on randomized controlled trials (RCTs), which are the gold standard for determining the effectiveness of interventions, in an attempt to provide a high-quality synthesis of available evidence. The review aims to assess VRER's effect on functional outcome, QoL, patient satisfaction, adherence, and safety in cancer survivors and hence inform clinical practice, guide rehabilitation program development, and shape future research on oncology-based digital health interventions.

**Methods**

**Study Design**

The systematic review was reported in accordance with the PRISMA 2020 reporting guideline [19]. The review protocol was prospectively registered in the International Prospective Register of Systematic Reviews (PROSPERO; ID: CRD42025637048). The major aim of the review was to synthesize the evidence from randomized controlled trials (RCTs) that compared the effects of virtual reality (VR)-based exercise therapy on cancer-related dysfunctions (CRDs) between adult patients and survivors. CRDs were understood as impairments in psychological, cognitive, or physical functioning because of cancer or its treatment.

**Search Strategy**

A systematic literature search was conducted in four major databases: PubMed, Scopus, Web of Science, and the Cochrane Central Register of Controlled Trials (CENTRAL) from inspection to November 2024. A combination of free-text terms and controlled vocabulary like MeSH terms has been utilized in the strategy for maximum sensitivity and specificity, and Boolean operators have been applied to filter the results. PubMed search term was the following: (((("Neoplasms"[MeSH Terms] OR cancer OR tumor OR oncology OR malignancy) AND (("Virtual Reality"[MeSH Terms] OR virtual reality OR immersive reality OR VR OR augmented reality OR mixed reality OR exergaming OR video games OR serious games))) AND (("Rehabilitation"[MeSH Terms] OR physical therapy OR physiotherapy OR exercise therapy OR motor activity OR therapeutic exercise)) AND (("Randomized Controlled Trial"[Publication Type] OR randomized OR randomized OR RCT))). The same methods were applied to the other databases. Additional manual checking of included studies' reference lists and relevant systematic reviews was also undertaken to determine whether there were additional eligible trials.

**Eligibility Criteria**

Eligibility criteria were developed using the Population, Intervention, Comparator, Outcomes, and Study Design (PICOS) system. The population was adults aged 18 and above with cancer diagnosis at any stage or treatment who also had one or more CRDs. The intervention was VR-based exercise or rehabilitation provided using immersive, semi-immersive, or non-immersive platforms to enhance physical, cognitive, or psychological outcomes. The comparator was traditional rehabilitation, usual care, sham VR, or no intervention. The primary outcomes were muscle strength, range of motion, functional status, quality of life, and physical performance, and secondary outcomes were adverse events and other related clinical or psychological outcomes. Only English-published randomized controlled trials were included. Studies were excluded if non-randomized, observational, case reports, protocols, reviews, included a pediatric population, or involved cancer unrelated conditions.

**Data Extraction**

Separate extraction of data by two reviewers was done using a standard form for precision and minimizing bias. Extracted data included study features such as author, year, and country, participant information such as sample size, mean age, sex split, type of cancer, and treatment status, data on the intervention such as type of VR system, frequency, duration, and delivery mode, comparator details, outcome measures, follow-up time, and main findings. Resolution of any discrepancies among the reviewers was either by discussion or referral to a third reviewer.

**Risk of Bias Assessment**

Two reviewers independently assessed the methodological quality of included RCTs with the aid of the Cochrane Risk of Bias 2 (ROB2) tool [20]. This involved evaluating the randomization process, deviations from the intended treatment, completing outcome data, validity and reliability of outcome measures, and selection of the reported outcomes. All the domains were given low risk, some issues, or high risk of bias, and disputes were resolved by discussion or by a third reviewer as required.

**Data Synthesis**

Due to heterogeneity across VR modalities, intervention protocols, study groups, and outcome measures, a meta-analysis could not be performed. Therefore, a narrative synthesis approach was used to synthesize and interpret findings. Synthesis was reported within outcome domains, including strength and range of motion, functional status, quality of life, physical performance, and adverse events, in order to enable organized interpretation of the evidence across the included trials.

**Results**

**Study selection and included trials**

The systematic search yielded 1,245 records, and 27 full-text articles were screened, with 8 RCTs included (Figure 1). The trials represented a total of 359 adult cancer survivors [21–28]. Cancer populations were heterogeneous and comprised breast cancer [21,22,25], prostate cancer [26], brain tumor [23], and mixed cancer cohorts [24,27,28]. Sample sizes ranged between 24 and 72 participants. Intervention durations varied widely from 2 to 12 weeks, at a frequency of 2 to 5 sessions per week.

VR-based rehabilitation interventions were heterogeneous: Kinect-based exergaming [22,25,26], immersive cognitive-motor VR environments [24], augmented reality telerehabilitation [21], a Virtual Therapeutic Garden [23], game-based balance training [27], and combined VR–exercise interventions [28]. Comparators included usual care [21,29], home-based unsupervised exercise [21,26], and conventional physiotherapy programs [22,25,27,28]. Outcomes were classified into five general domains: (1) strength and range of motion (ROM), (2) functional status, (3) physical performance, (4) health-related quality of life (QoL), and (5) safety/adverse events (Table 1).

**Identification of studies via databases and registers**

Records identified from*:

PubMed (n= 225),

Scopus (n=322),

Web of Science (n=225) and Cochrane (n = 161)

Records removed *before screening*:

Duplicate records removed (n = 363)

Records marked as ineligible by automation tools (n= 205)

**Identification**

Records excluded:

irrelevant titles and abstracts

(n = 391)

population not eligible, outcome mismatch, ineligible study design

(n = 130)

Records screened

(n = 570)

**Screening**

Reports excluded: (n=41)

Reports assessed for eligibility

(n = 49)

Studies included in review

(n =8)

**Included**

*Figure 1 \ flow chart for study selection process*

*Table 2 / characteristics of included study*

| Results | Outcome description | Control | intervention | participants | Design | Study ID | s |
| --- | --- | --- | --- | --- | --- | --- | --- |
| better improvement in the VR group than in the control group in terms of (K-MBI) index. | the K-MBI were used to evaluate cognitive function and functional status. Patients were evaluated pre and post intervention (week 4). | computer-assisted cognitive rehabilitation 5 times a week for 30 minutes for a total of 4 weeks. | VR group (n=19) received VR training 3 times a week for 30 minutes and computer-assisted cognitive rehabilitation 2 times a week for 30 minutes for a total of 4 weeks. | 38 brain tumor patients (19 men and 19 women) >20 years old with cognitive impairment, who had received treatments, such as surgery, radiotherapy, or chemotherapy. | RCT | Yang et al ,2014 [23] | 1 |
| greater improvements in shoulder, elbow and forearm function in the intervention group and hand function in the control group. | upper extremity muscle power and function via Box and Block test, Manual Function test, Fugl-Meyer scale | conventional occupational therapy alone 30 mins per session for 15 sessions for 3 wks. | virtual reality program 30 mins per session for 9 sessions and conventional occupational therapy 30 mins per session for 6 sessions for 3 weeks, | 20 patients aged between 40 to 60 years diagnosed with brain tumor after proper management | RCT | Yoon et al, 2015 [28] | 2 |
| Training was safe and well accepted despite participants’ impaired health status., sway of hip, ankle, and center of mass were significantly reduced in the intervention group compared to control group during standing in feet-close-position with eye open (p < 0.001). while in eye closed, they did not achieve statistical significance | balance including changes in sway of ankle, hip, and COM in both ML and AP directions during 30-second balance tests with increasing task difficulty | no exercise intervention and continued their normal activity. | interactive game-based balance training including repetitive weight shifting and virtual obstacle crossing task. two sessions per week (45 min. each), for four weeks | 22 22 patients (age: 70.3 ± 8.7 years) with current or prior malignancy (lung, breast, MM, breast, colorectal, prostate, pancreas, melanoma, ovarian, CLL) who were receiving chemotherapy | RCT | Schwenk et al,2016 [27] | 3 |
| significant improvement in the exergaming group compared to the usual care (p < 0.001). While there was no significant difference in other outcomes between intervention and control groups | 6MWT composition (lean- and fat-mass), (EORTC QLQ-C30), Quality of Life (FACT-P) and - (FACT-F). | Usual Care | 12 weeks of unsupervised home-based exergaming | 46 patients with prostate cancer (aged >65 years) with locally advanced or advanced stage disease undergoing ADT | RCT | Villumsen et al,2019 [26] | 4 |
| Results | Outcome description | Control | intervention | participants | Design | Study ID | s |
| Statistically significant improvements (p < 0.001) were observed in both groups across all measured outcomes. The Xbox Kinect group showed greater reductions in fear of movement, while the standardized physiotherapy group demonstrated significantly greater improvements in upper limb functionality. | Pain, grip strength, functionality, muscle strength, ROM, and fear of movement. The outcomes were evaluated pre and post intervention (6 weeks). | conventional upper extremity physiotherapy program | Kinect-Based Rehabilitation Group + conventional physical therapy program (n = 20) | 40 women, aged 30–60 years, in the second postoperative week following breast cancer surgery with axillary dissection. | RCT | Feyzioğlu et al ,2020 [22] | 5 |
| Statistically significant differences were recorded in shoulder ROM, and general health, in favor of the Xbox Kinect group (p < 0.001). However, there were statistically significant differences in shoulder flexion strength, external rotation strength, and abduction strength and handgrip strength in favor of the resistance exercise group (p < 0.001). | shoulder ROM, shoulder muscles strength, hand grip strength, and Study (SF-36). The outcomes were evaluated pre and post intervention (week 8). | resistance exercise group received resistance training (n = 30) + complex decongestive physiotherapy (manual lymphatic drainage, compression bandages, skin care, and | Xbox Kinect group received VR Kinect-based games (n = 30) + complex decongestive physiotherapy (manual lymphatic drainage, compression bandages, skin care, and | 60 women with unilateral breast cancer related lymphedema, Ages: >30 years old. | Single-blinded RCT | Basha et al, 2022 [25] | 6 |
| In both groups, active and passive shoulder ROM, and the QuickDASH, FACT-B, and EQ5D-5L scores showed significant improvements by the end of treatment (p < 0.001), but no group differences were recorded. | Shoulder range of motion, functional outcomes, and quality of life. Outcomes were measured at enrollment and after 4, 8, and 12 weeks | Brochure-based rehabilitation group (n=50) received a brochure detailing the same exercise program used for the intervention group. Daily exercise was performed for 8 weeks. | AR-based digital health care system group (n=50) received telerehabilitation by accessing the prescribed daily exercises through the internet receiving instant feedback through sensors. Daily exercise was performed for 8 weeks. | 100 women aged 20–70 who had undergone axillary lymph node dissection or breast reconstruction following mastectomy | RCT | Park et al ,2023 [21] | 7 |
| Significant differences favoring the EG were identified in the group x time interactions for the main outcome (p < 0.001) | IPAQ | passive control | 2 weeks of Virtual Therapeutic Garden. 15 min for the session for eight sessions | 33 patients (age55 to60) diagnosed with breast cancer regardless of the disease stage the patient’s age. | RCT | Czech et al ,2024 [24] | 8 |

K-MBI: Korean Modified Barthel Index, MM: multiple myeloma, CLL: chronic lymphoid leukemia COM: Center of Mass, ML: Mediolateral, AP: Anteroposterior, 6MWT: 6 minute walk test, LEP: leg extensor power, EORTC QLQ-C30: self-reported physical functioning and global health status, FACT-P/FACT-F: Functional Assessment of Cancer Therapy – Prostate/fatigue, ROM: Range of Motion, SF-36: Short-Form questionnaire, QuickDASH: Disabilities of the Arm, Shoulder and Hand questionnaire, EQ5D-5L: EuroQol five-dimensional five-level questionnaire, IPAQ: physical activity via International physical activity questionnaire, EG: experimental group.

**Risk of bias (ROB2) assessment**

The risk of bias was evaluated using the Cochrane ROB2 tool (Figure 2). Four trials were rated as overall low risk [21, 24]. They showed adequate randomization, allocation concealment, and complete outcome reporting. Two studies raised some concerns, mainly because of insufficient details on randomization and incomplete data management [25, 26]. The last two studies were considered high risk [27, 28]. This was due to selective reporting, a lack of pre-registered protocols, and imbalances between groups at baseline. When interpreting the evidence, the results from low-risk trials were given more weight. Findings from studies with higher bias were approached with caution.

*Figure 2 \ Risk of bias assessment for included studies*

**Characteristics of Virtual Reality-Based Exercise Rehabilitation**

**Strength and Range of Motion**

Three RCTs assessed shoulder range of motion (ROM) and muscle strength [21, 22, 25]. Park et al. [21] showed significant improvements in shoulder flexion (mean difference [MD]: 12.3°, 95% CI: 7.5–17.1, p<0.001) and abduction (MD: 11.8°, 95% CI: 6.1–17.5, p<0.001) after 8 weeks of VR-based telerehabilitation compared to brochure-based home exercise. Feyzioğlu et al. [22] found that Kinect-based VR led to ROM and pain outcomes similar to supervised physiotherapy (p>0.05). Basha et al. [25] reported that while VR resulted in functional improvement, traditional resistance-based therapy achieved better specific gains in shoulder flexion, external rotation, and grip strength. Overall, VR consistently improved ROM and reduced fear of movement, although purpose-specific resistance exercise was superior for isolated strength outcomes.

**Functional Status (Activities of Daily Living)**

Four studies looked at activities of daily living (ADL) and functional independence [21, 23, 26, 28]. Park et al. [21] noted meaningful improvements in the Functional Assessment of Cancer Therapy–Breast (FACT-B) physical well-being scores. Navarro-López et al. [23], using the Virtual Therapeutic Garden, showed improved self-care and cognitive-motor integration in brain tumor survivors (p<0.05). Villumsen et al. [26] found significant improvements in the 6-minute walk distance (MD: 21.5 m; 95% CI: 3.2–39.9; p=0.023) in prostate cancer patients after 12 weeks of exergaming. Meanwhile, Lo Presti et al. [28] reported increased autonomy in daily activities following VR balance training. Overall, VR interventions improved endurance, independence, and functionality across different cancer groups.

**Physical Performance and Exercise Capacity**

Three RCTs evaluated physical performance outcomes [24, 26, 27]. Czech et al. [24] found that immersive VR therapy increased self-reported moderate-intensity physical activity and reduced sedentary time. Villumsen et al. [26] showed improvements in aerobic capacity with longer walking distances in the 6-minute walk test (6MWT). Schwenk et al. [27] demonstrated that VR-based balance training significantly reduced postural sway at the hip, ankle, and center of mass during challenging stance tasks (p=0.008–0.035). Together, these findings suggest that VR effectively improves endurance, balance control, and functional exercise tolerance, even among cancer survivors with health issues.

**Quality of Life (QoL)**

Three trials assessed health-related quality of life [21, 25, 26]. Park et al. [21] showed clinically relevant improvement in FACT-B physical well-being (MD: 3.5; 95% CI: 1.2–5.8; p<0.01). Basha et al. [25] reported significant improvements in SF-36 bodily pain and general health subscales (p<0.01) following VR rehabilitation. Villumsen et al. [26] indicated positive changes in vitality and general health dimensions. These findings suggest that VR interventions can improve both physical and psychosocial aspects of survivorship. In addition, two studies examined psychological dimensions [22, 23]. Feyzioğlu et al. [22] observed reductions in fear of movement (Tampa Scale of fear of movement) like conventional physiotherapy (p<0.05). Navarro-López et al. [23] reported VR-related improvements in cognitive-motor function, attention, and motivation. This contributed to better adherence and engagement. These results highlight VR’s dual role in meeting both physical and psychological rehabilitation needs.

**Adverse Events and Tolerability**

Two RCTs systematically reported adverse events [22, 27]. No serious adverse events occurred. Mild, temporary symptoms like dizziness and muscle soreness were reported occasionally but resolved on their own. Adherence rates exceeded 85% in all studies, and no trial required discontinuation due to safety issues. However, the limited monitoring across studies restricts conclusions about rare or long-term adverse effects.

**Synthesis Across Domains and Certainty Considerations**

In the eight RCTs, the most consistent and clinically significant benefits of VR-based rehabilitation were observed in shoulder mobility, balance control, and functional capacity. Improvements in quality of life and psychological outcomes were reported but were less consistent, influenced by differences in intervention design, cancer populations, and outcome measures. High adherence and a lack of major safety concerns support the feasibility of VR interventions in oncology rehabilitation. Evidence from low-risk trials [21–24] strongly supports VR's effectiveness in improving range of motion and functional capacity. Results from trials with some concerns or high risk [25–28] need cautious interpretation. Methodological differences, including intervention intensity, technology type, and control conditions, prevented a meta-analysis but allowed for a robust narrative synthesis. These findings underscore VR's potential as a complementary rehabilitation approach while highlighting the need for larger, standardized RCTs with consistent outcome measures to strengthen the evidence base.

**Discussion**:

The present systematic review synthesized evidence from eight randomized controlled trials that evaluated the efficacy of virtual reality (VR)-based exercise interventions for cancer-related dysfunctions (CRDs) including strength, range of motion, functional status, quality of life, physical performance, and safety. The included trials involved diverse cancer types and rehabilitation settings, suggesting that VR interventions may be effective across a wide range of patients and contexts. Overall, evidence shows that VR is an effective and safe addition to conventional rehabilitation, with regular physical and functional gains and favorable safety outcomes. This result aligns with results in neurological and orthopedic rehabilitation, where interactive, immersive, and task-oriented VR methods enhanced neuroplasticity, compliance, and functional outcome [29,30].

**Strength and Joint Range of Motion**

Four trials tested the effects of VR-based rehabilitation on muscle strength and upper-limb range of motion, specifically in the shoulder and hand [21,22,25,28]. Across trials, VR demonstrated positive effects on isometric strength and functional mobility compared with usual care. These effects can be attributed to the interactive and task-oriented nature of VR programs to facilitate repetitive, goal-directed movement in a safe and engaging environment. in a mechanical way, VR enhances neuromuscular adaptation, proprioceptive feedback, and motor unit recruitment, all of which are consistent with neurorehabilitation evidence [31,32]. The positive effect on ROM can be explained by VR's implementation of gradual joint mobilization into functional tasks in a manner that reduces fear of movement and optimizes compliance. This is consistent with evidence from stroke, where VR improved active shoulder mobility using graded exposure and practice repetition [29]. Availability of real-time auditory and visual feedback also promoted even higher motor learning and reduced compensatory strategy use, for the betterment of functional improvement. Importantly, these benefits were achieved with no increase in adverse events, confirming the safety of VR as a musculoskeletal rehabilitation program in cancer treatment.

**Functional Status**

Four randomized controlled trials evaluated the effect of VR-based rehabilitation on functional status and ability to perform activities of daily living (ADLs) [21,23,26,28]. During these trials, VR produced large gains in function compared to physiotherapy, usual care, or rehabilitation alone. The gains were attributable to the capacity of VR to simulate realistic rehearsal of daily life skills within a controlled, progressively demanding environment, which facilitates the transfer and acquisition of skills. In terms of Neurophysiology, immersive VR potentially amplifies cortical reorganization and motor planning, supplementing functional recovery in cancer patients [33]. The interactive nature of VR also provides problem-solving, sequencing, and multisensory stimulation that engages both cognitive and motor systems simultaneously. Multimodal stimulation is particularly useful in oncology patients where fatigue, neuropathy, and deconditioning due to treatment can limit recovery potential. Similar results were observed in other fields of rehabilitation such as stroke and geriatrics where interventions based on VR enhanced independence and reduced the need for caregivers [34,35]. Importantly, no adverse events occurred during ADL-focused VR trials, validating the safety and feasibility of using VR for augmenting functional independence in cancer rehabilitation.

**Physical Performance**

Three randomized controlled trials tested the effect of VR-based rehabilitation on objective measures of physical performance, including gait, balance, and overall physical activity [24,26,27]. Czech 2024 [24] found improvements in level of physical activity after two weeks of high-intensity "Virtual Therapeutic Garden" use, implying that nature-based VR environments can raise motivation and engagement following treatment for cancer. Schwenk 2016 [27] demonstrated clinically significant reductions in postural sway during balance tasks in patients with chemotherapy-induced neuropathy, suggesting that VR may improve postural control even in medically frail patients. Villumsen 2019 [26] determined that home-based unsupervised exergaming enhanced six-minute walk distance and leg strength in prostate cancer patients, affirming VR's capacity to stimulate endurance and strength adaptation that is equivalent to supervised exercise. These findings are consistent with neurorehabilitation literature, in which VR-based gait and balance training improves mobility and stability via task-specific repetition, augmented feedback, and multisensory engagement [37]. Immersion in VR may also enhance neuroplasticity through motor imagery and action observation, mechanisms of particular interest in oncology patients undergoing treatment-related deconditioning. Although heterogeneity of intervention type, duration, and outcome measures limits firm conclusions regarding optimal parameters, excellent adherence was documented by all three studies, which suggests that VR-based performance gains are possible and maintainable in cancer rehabilitation.

**Quality of Life (QoL):**

Three randomized controlled trials assessed the impact of VR-based rehabilitation on health-related quality of life in adult cancer survivors, registering clinically important improvements, notably in physical functioning, vitality, and global health [22,25,26]. These gains appeared to be the result of the combined influence of structured physical activity, feedback immediately provided, and the interactive quality of gamified VR tasks, which provide motivation, reduce perceived exertion, and facilitate self-efficacy. For example, VR arms in Basha 2022 [25] and Park 2023 [22] reported similar or larger benefits compared to active controls such as usual physiotherapy or brochure-based exercise, highlighting value added from immersion and interactivity over and above exercise content. In prostate cancer patients, Villumsen 2019 [26] showed that unsupervised home-based exergaming improved overall health and physical functioning above standard care, proving effective remote rehabilitation application of VR. this aligns with oncology evidence for exercise as a cause of reduced fatigue, improved mood, and improved functional capacity, pathways further enhanced by VR's reward loops and progressive challenge design [36]. But heterogeneity across measurement instruments (SF-36, FACT-B, FACT-P, EORTC QLQ-C30, EQ-5D-5L), intervention duration, and comparators prevented cross-trial comparability. Though some individual domains were not statistically significant, the direction of effect was consistently positive, and no trial reported health related quality of life worsening, confirming the clinical value and acceptability of VR interventions to be utilized in survivorship care.

**Adverse Events:**

Two randomized controlled trials comprehensively evaluated adverse events during VR-based rehabilitation and confirmed that the treatments were safe and well tolerated [22,27]. Among breast cancer survivors, no intervention-related complications of lymphedema worsening or musculoskeletal trauma were observed when Kinect-based training was compared with standard physiotherapy by Feyzioğlu 2020 [22]. Neither falls nor severe adverse events were experienced among oncology patients on chemotherapy, even after performing demanding balance exercises, according to Schwenk 2016 [27]. Transient mild complaints such as muscle soreness, fatigue, or dizziness occasionally occurred but spontaneously disappeared without therapy interruption. These findings are consistent with the broader rehabilitation literature in validating VR as a safe method of treatment for orthopedic and neurological populations [38]. Overall, the evidence suggests that VR can be safely incorporated into oncology rehabilitation with interventions matched to levels of patient function and delivered under adequate supervision.

**Types of Virtual Reality Interventions and Their Effects on Outcomes**

The RCTs summarized here used diverse VR modalities, ranging from fully immersive head-mounted display (HMD) systems [24,27] to non-immersive screen-based exergaming systems [21,22], with some trials using semi-immersive projection-based systems. Immersive VR had stronger engagement and sensory feedback, and this may explain why those trials yielded greater increases in physical activity, balance, and psychological outcomes. Non-immersive systems, in contrast, were associated with less complexity to implement, lower cost, and minimal risk for cybersickness but still with the impressive gains in joint mobility and functional status. Semi-immersive modalities, although less common, provide accessibility with some sensory presence and can reduce fatigue without compromising on therapeutic stimulation. Importantly, efficacy did not generalize to a single modality, with both immersive and non-immersive systems proving advantageous on both functional and psychosocial outcomes. These findings highlight the need for VR modality to be tailored to patient profile, therapeutic goals, and the resources available so that interventions are both effective and feasible in oncology rehabilitation.

**Comparison with Existing Literature:**

These review results are largely consistent with outcomes of VR use in neurological, musculoskeletal, and geriatric rehabilitation, where VR was seen to improve motor function, range of motion, and activities of daily living [38,39,40]. In cancer, underlying mechanisms appear also to include recovery, with VR supporting musculoskeletal retraining, reducing treatment-related fatigue, and improving psychosocial stimulation via interactive environments. Quality of life improvements cited herein are consistent with those reported in non-cancer VR meta-analyses [14], where motivation, engagement, and compliance were primary drivers of benefit. However, heterogeneity in intervention duration and cancer issues such as chemotherapy-induced neuropathy, anemia, or cardiopulmonary instability may affect outcomes and require more VR protocol adjustment. Physical improvements reported in Czech 2024 [24] and Schwenk 2016 [26] also corroborate current evidence from VR-based fall prevention programs in older adults [41], emphasizing the transferability of VR motor learning principles to various populations. Safety outcomes were in keeping with more general rehabilitation literature, with little risk of harm in individualized, supervised programs [42]. Overall, the advantages of VR are in line with current rehabilitation evidence, but oncology-specific factors suggest the benefit of tailoring interventions by cancer diagnosis, treatment stage, and functional status to optimize outcomes.

**Strengths of the Review:**

There are several strengths of this systematic review that enhance the validity and clinical relevance of its findings. First, it adhered to PRISMA 2020 guidelines and was prospectively registered in PROSPERO, reducing the risk of reporting bias and methodological transparency. Second, only randomized controlled trials were considered, which increases internal validity compared to earlier reviews that had included non-randomized designs as well. Third, the search strategy was comprehensive, covering four main databases and supplemented by manual screening of references to minimize the likelihood of excluding relevant studies. Finally, data extraction and risk-of-bias were conducted independently by two reviewers with the assistance of the Cochrane ROB2 tool in order to maximize objectivity as well as methodological rigor. Importantly, this review is the first to focus exclusively on VR-based exercise interventions for cancer dysfunctions and includes evidence of direct relevance to oncology rehabilitation and survivorship care planning.

**Limitations**:

This review has some important limitations that should be noted. First, the included randomized controlled trials (RCTs) varied widely in terms of cancer type, disease stage, virtual reality (VR) method, frequency of intervention, and duration of programs. This variability made it hard to perform a meta-analysis, so we had to rely on a narrative synthesis. While this has its value, it weakens the overall strength of the evidence. Additionally, the outcome measures used in the trials were very different. For example, assessments of functional status, fatigue, and quality of life employed various scales and tools. This diversity made comparisons difficult and complicated efforts to create unified clinical recommendations.

The methodological issues in the included trials further limit the evidence gathered. Blinding of participants and therapists was often impossible in most VR interventions, which introduced a risk of performance bias. Although blinding of outcome assessors could have reduced this risk, information about assessor blinding was frequently incomplete. Another challenge was the small sample sizes and brief intervention periods in many trials. These issues not only reduced statistical power but also made it hard to draw strong conclusions about the long-term benefits of VR in cancer survivorship.

Finally, even though there were no serious adverse events reported, safety monitoring was applied inconsistently, and reporting of adverse events was limited. This raises concerns about possible underreporting, making it hard to definitively say that VR-based rehabilitation has no risks. In summary, while the current findings are encouraging, they should be viewed with caution until larger RCTs with more consistent methods and longer follow-up are conducted.

**Recommendations for Clinical Implications and Future Directions**

VR-exercise therapy interventions are highly likely to be utilized as an adjunct to conventional oncology rehabilitation and show physical, psychological, and cognitive advantages with excellent patient motivation and adherence. Clinically, the interventions must be individualized based on factors such as the cancer type, treatment stage, functional capacity, and psychosocial issues. Immersive VR may have the potential to provide greater motivational and cognitive advantages, yet non-immersive technology remains cost-effective and more practicable in resource-constrained environments [43]. Such combination with multimodal rehabilitation regimens, including resistance and aerobic exercise, may enhance outcomes, if patient safety is closely monitored.

Subsequent studies will have to be large-scale, multicenter RCTs with protocol standardization including VR modality, dose, and criteria for advancing. Follow-up over the long term is important to evaluate sustainability of the benefits, while mechanistic studies employing biomarkers or neuroimaging would help explain underlying mechanisms [44]. In addition, economic evaluations and subgroup analyses by sex, age, stage of treatment, and type of cancer are needed to guide implementation and policy. Collectively, these steps will enable integration of VR into routine oncology rehabilitation and enhance recovery and survivorship care.

**Conclusion**

This systematic review synthesizes evidence from eight randomized controlled trials demonstrating virtual reality (VR)–based exercise therapy can offer substantial benefits in cognitive domains for patients with cancer-related physical, psychological, and to some extent, cognitive dysfunctions. The outcomes demonstrate improvements in upper limb strength, range of motion, functional ability, physical functioning, and overall quality of life with minimal adverse events reported, highlighting the safety and applicability of VR interventions within heterogeneous oncology populations.

Despite heterogeneity of VR modalities, treatment length, intensity, and outcome measures, global evidence consistently supports VR-based rehabilitation as a valuable adjunct to conventional therapy. The positive effects reported across various domains suggest that VR can engage patients, provide interactive and inspiring experience, and perhaps solve complex rehabilitation dilemmas in oncology settings.

High-integrity, large-scale randomized controlled trials with standardized protocols, clearly defined intervention parameters, and extended follow-up periods must be conducted in the future to confirm these effects. These trials must identify optimal VR strategies, customize treatment dosage and intensity, and guide the translation of VR-based interventions into routine clinical oncology practice. Bridging these gaps, VR-based exercise therapy can become an applied, safe, and evidence-based component of integrative cancer rehabilitation programs.

**Funding**

The authors declare that no funds, grants, or other financial support were received during the preparation of this manuscript.

**Competing Interests**

The authors have no relevant financial or non-financial interests to disclose.

**Author Contributions**

Manar Eid, Haytham Alkerdasy, Abdelrahman Sameh, Amina Awad, and Amal Ashraf contributed to the study design, data collection, and analysis. The manuscript was drafted by Manar Eid and revised by co-authors. All authors approved the final version of the manuscript

**Data Availability**

All data generated or analyzed during this study are included in this published article. Additional details are available from the corresponding author on reasonable.

**References**

1. Battaglini CL, Hackney AC, Garcia R, Groff D, Evans E, Shea T. The effects of an exercise program in leukemia patients. Integr Cancer Ther. 2009 Jun;8(2):130-8. doi: 10.1177/1534735409334266. PMID: 19679621.
2. Courneya KS, Friedenreich CM. Physical activity and cancer control. Semin Oncol Nurs. 2007 Nov;23(4):242-52. doi: 10.1016/j.soncn.2007.08.002. PMID: 18022052.
3. Joly F, Lange M, Dos Santos M, Vaz-Luis I, Di Meglio A. Long-Term Fatigue and Cognitive Disorders in Breast Cancer Survivors. Cancers (Basel). 2019 Nov 28;11(12):1896. doi: 10.3390/cancers11121896. PMID: 31795208; PMCID: PMC6966680.
4. Stout NL, Santa Mina D, Lyons KD, Robb K, Silver JK. A systematic review of rehabilitation and exercise recommendations in oncology guidelines. CA Cancer J Clin. 2021 Mar;71(2):149-175. doi: 10.3322/caac.21639. Epub 2020 Oct 27. PMID: 33107982; PMCID: PMC7988887.
5. Dennett AM, Peiris CL, Shields N, Prendergast LA, Taylor NF. Moderate-intensity exercise reduces fatigue and improves mobility in cancer survivors: a systematic review and meta-regression. J Physiother. 2016 Apr;62(2):68-82. doi: 10.1016/j.jphys.2016.02.012. Epub 2016 Mar 17. PMID: 26996098.
6. Addison, S., Shirima, D., Aboagye-Mensah, E.B. *et al.* Effects of tandem cognitive behavioral therapy and healthy lifestyle interventions on health-related outcomes in cancer survivors: a systematic review. *J Cancer Surviv* **16**, 1023–1046 (2022). <https://doi.org/10.1007/s11764-021-01094-8>
7. Nizeyimana E, Joseph C, Plastow N, Dawood G, Louw QA. A scoping review of feasibility, cost, access to rehabilitation services and implementation of telerehabilitation: Implications for low- and middle-income countries. *DIGITAL HEALTH*. 2022;8. doi:[10.1177/20552076221131670](https://doi.org/10.1177/20552076221131670)
8. Watts, T., Courtier, N., Fry, S. *et al.* Access, acceptance and adherence to cancer prehabilitation: a mixed-methods systematic review. *J Cancer Surviv* (2024). https://doi.org/10.1007/s11764-024-01605-3
9. Wu Y, Wang N, Zhang H, Sun X, Wang Y, Zhang Y. Effectiveness of Virtual Reality in Symptom Management of Cancer Patients: A Systematic Review and Meta-Analysis. J Pain Symptom Manage. 2023 May;65(5):e467-e482. doi: 10.1016/j.jpainsymman.2023.01.023. Epub 2023 Feb 8. PMID: 36758907.
10. Rutkowski, Sebastan, Luisa Cacciante, Joanna Szczepańska-Gieracha, Błażej Cieślik, Pawel Kiper, Andrea Turolla, and Justyna Mazurek. 2020. “Use of Virtual Reality-Based Training in Different Fields of Rehabilitation: A Systematic Review and Meta-Analysis.” Journal of Rehabilitation Medicine 52 (11). Medical Journals Sweden Ab: jrm00121. doi:10.2340/16501977-2755.
11. Villumsen BR, Jorgensen MG, Frystyk J, Hørdam B, Borre M. Home-based 'exergaming' was safe and significantly improved 6-min walking distance in patients with prostate cancer: a single-blinded randomised controlled trial. BJU Int. 2019 Oct;124(4):600-608. doi: 10.1111/bju.14782. Epub 2019 May 14. PMID: 31012238.
12. Burrai F, Sguanci M, Petrucci G, De Marinis MG, Piredda M. Effectiveness of immersive virtual reality on anxiety, fatigue and pain in patients with cancer undergoing chemotherapy: A systematic review and meta-analysis. Eur J Oncol Nurs. 2023 Jun; 64:102340. doi: 10.1016/j.ejon.2023.102340. Epub 2023 May 16. PMID: 37290160.
13. Tough D, Robinson J, Gowling S, Raby P, Dixon J, Harrison SL. The feasibility, acceptability and outcomes of exergaming among individuals with cancer: a systematic review. BMC Cancer. 2018 Nov 21;18(1):1151. doi: 10.1186/s12885-018-5068-0. PMID: 30463615; PMCID: PMC6249900.
14. Hao J, Li Y, Swanson R, Chen Z, Siu KC. Effects of virtual reality on physical, cognitive, and psychological outcomes in cancer rehabilitation: a systematic review and meta-analysis. Support Care Cancer. 2023 Jan 12;31(2):112. doi: 10.1007/s00520-022-07568-4. PMID: 36633695.
15. Mao W, Chen W, Wang Y. Effect of virtual reality-based mindfulness training model on anxiety, depression, and cancer-related fatigue in ovarian cancer patients during chemotherapy. Technol Health Care. 2024;32(2):1135-1148. doi: 10.3233/THC-230735. PMID: 37781832; PMCID: PMC11002720.
16. Filakova K, Janikova A, Felsoci M, Dosbaba F, Su JJ, Pepera G, Batalik L. Home-based cardio-oncology rehabilitation using a telerehabilitation platform in hematological cancer survivors: a feasibility study. BMC Sports Sci Med Rehabil. 2023 Mar 23;15(1):38. doi: 10.1186/s13102-023-00650-2. PMID: 36959613; PMCID: PMC10034898.
17. Su Z, Zhang L, Lian X, Guan M. Virtual Reality-Based Exercise Rehabilitation in Cancer-Related Dysfunctions: Scoping Review. J Med Internet Res. 2024 Feb 26;26:e49312. doi: 10.2196/49312. PMID: 38407951; PMCID: PMC10928524.
18. Zhang H, Xu H, Zhang ZX, Zhang Q. Efficacy of virtual reality-based interventions for patients with breast cancer symptom and rehabilitation management: a systematic review and meta-analysis. BMJ Open. 2022 Mar 17;12(3):e051808. doi: 10.1136/bmjopen-2021-051808. PMID: 35301201; PMCID: PMC8932270.
19. Page MJ, McKenzie JE, Bossuyt PM, Boutron I, Hoffmann TC, Mulrow CD, Shamseer L, Tetzlaff JM, Akl EA, Brennan SE, Chou R. The PRISMA 2020 statement: an updated guideline for reporting systematic reviews. bmj. 2021 Mar 29;372. doi: [10.1136/bmj.n71](https://doi.org/10.1136/bmj.n71). PMCID: [PMC8005924](https://pmc.ncbi.nlm.nih.gov/articles/PMC8005924/)
20. Higgins JP, Sterne JA, Savovic J, Page MJ, Hróbjartsson A, Boutron I, Reeves B, Eldridge S. A revised tool for assessing risk of bias in randomized trials. Cochrane database of systematic reviews. 2016;10(Suppl 1):29-31. doi: [10.1136/bmj.l4898](https://doi.org/10.1136/bmj.l4898). PMID: 31462531
21. Park HY, Nam KE, Lim JY, Yeo SM, In Lee J, Hwang JH. Real-time interactive digital healthcare system for post-operative breast cancer patients: study protocol for a randomized controlled trial. Trials. 2021 Aug 19;22(1):549. doi: 10.1186/s13063-021-05535-8. PMID: 34412658; PMCID: PMC8374413.
22. Feyzioğlu Ö, Dinçer S, Akan A, Algun ZC. Is Xbox 360 Kinect-based virtual reality training as effective as standard physiotherapy in patients undergoing breast cancer surgery? Support Care Cancer. 2020 Sep;28(9):4295-4303. doi: 10.1007/s00520-019-05287-x. Epub 2020 Jan 6. PMID: 31907649.
23. Yang S, Chun MH, Son YR. Effect of virtual reality on cognitive dysfunction in patients with brain tumor. Ann Rehabil Med. 2014 Dec;38(6):726-33. doi: 10.5535/arm.2014.38.6.726. Epub 2014 Dec 24. PMID: 25566470; PMCID: PMC4280367.
24. Czech O, Kowaluk A, Ściepuro T, Siewierska K, Skórniak J, Matkowski R, Malicka I. Effects of Immersive Virtual Therapy as a Method Supporting the Psychological and Physical Well-Being of Women with a Breast Cancer Diagnosis: A Randomized Controlled Trial. Curr Oncol. 2024 Oct 21;31(10):6419-6432. doi: 10.3390/curroncol31100477. PMID: 39451781; PMCID: PMC11505915.
25. Basha MA, Aboelnour NH, Alsharidah AS, Kamel FH. Effect of exercise mode on physical function and quality of life in breast cancer-related lymphedema: a randomized trial. Support Care Cancer. 2022 Mar;30(3):2101-2110. doi: 10.1007/s00520-021-06559-1. Epub 2021 Oct 20. PMID: 34669036.
26. BR, Jorgensen MG, Frystyk J, Hørdam B, Borre M. Home-based 'exergaming' was safe and significantly improved 6-min walking distance in patients with prostate cancer: a single-blinded randomised controlled trial. BJU Int. 2019 Oct;124(4):600-608. doi: 10.1111/bju.14782. Epub 2019 May 14. PMID: 31012238.
27. Schwenk M, Grewal GS, Holloway D, Muchna A, Garland L, Najafi B. Interactive Sensor-Based Balance Training in Older Cancer Patients with Chemotherapy-Induced Peripheral Neuropathy: A Randomized Controlled Trial. Gerontology. 2016;62(5):553-63. doi: 10.1159/000442253. Epub 2015 Dec 18. PMID: 26678611; PMCID: PMC6644035.
28. Yoon J, Chun MH, Lee SJ, Kim BR. Effect of virtual reality-based rehabilitation on upper-extremity function in patients with brain tumor: controlled trial. Am J Phys Med Rehabil. 2015 Jun;94(6):449-59. doi: 10.1097/PHM.0000000000000192. PMID: 25251249.
29. Suk, KS., Park, J., Choi, T. *et al.* Virtual reality-assisted rehabilitation for postoperative C5 palsy: a pilot exploratory randomized controlled trial. *J NeuroEngineering Rehabil* **22**, 178 (2025). <https://doi.org/10.1186/s12984-025-01716-7>
30. Levin MF, Weiss PL, Keshner EA. Emergence of virtual reality as a tool for upper limb rehabilitation: incorporation of motor control and motor learning principles. Phys Ther. 2015 Mar;95(3):415-25. doi: 10.2522/ptj.20130579. Epub 2014 Sep 11. PMID: 25212522; PMCID: PMC4348716.
31. Qian J, McDonough DJ, Gao Z. The Effectiveness of Virtual Reality Exercise on Individual's Physiological, Psychological and Rehabilitative Outcomes: A Systematic Review. Int J Environ Res Public Health. 2020 Jun 10;17(11):4133. doi: 10.3390/ijerph17114133. PMID: 32531906; PMCID: PMC7312871.
32. Holden MK. Virtual environments for motor rehabilitation: review. Cyberpsychol Behav. 2005 Jun;8(3):187-211; discussion 212-9. doi: 10.1089/cpb.2005.8.187. PMID: 15971970.
33. Mirelman A, Maidan I, Herman T, Deutsch JE, Giladi N, Hausdorff JM. Virtual reality for gait training: can it induce motor learning to enhance complex walking and reduce fall risk in patients with Parkinson's disease? J Gerontol A Biol Sci Med Sci. 2011 Feb;66(2):234-40. doi: 10.1093/gerona/glq201. Epub 2010 Nov 24. PMID: 21106702.
34. de Melo GEL, Kleiner AFR, Lopes JBP, Dumont AJL, Lazzari RD, Galli M, Oliveira CS. Effect of virtual reality training on walking distance and physical fitness in individuals with Parkinson's disease. NeuroRehabilitation. 2018;42(4):473-480. doi: 10.3233/NRE-172355. PMID: 29660956.
35. Matamala-Gomez M, Donegan T, Bottiroli S, Sandrini G, Sanchez-Vives MV, Tassorelli C. Immersive Virtual Reality and Virtual Embodiment for Pain Relief. Front Hum Neurosci. 2019 Aug 21;13:279. doi: 10.3389/fnhum.2019.00279. PMID: 31551731; PMCID: PMC6736618.
36. Meijer LL, Ruis C, van der Smagt MJ, Scherder EJA, Dijkerman HC. Neural basis of affective touch and pain: A novel model suggests possible targets for pain amelioration. J Neuropsychol. 2022 Mar;16(1):38-53. doi: 10.1111/jnp.12250. Epub 2021 May 12. PMID: 33979481; PMCID: PMC9290016.
37. Corbetta D, Imeri F, Gatti R. Rehabilitation that incorporates virtual reality is more effective than standard rehabilitation for improving walking speed, balance and mobility after stroke: a systematic review. J Physiother. 2015 Jul;61(3):117-24. doi: 10.1016/j.jphys.2015.05.017. Epub 2015 Jun 18. PMID: 26093805.
38. Dockx K, Bekkers EM, Van den Bergh V, Ginis P, Rochester L, Hausdorff JM, Mirelman A, Nieuwboer A. Virtual reality for rehabilitation in Parkinson's disease. Cochrane Database Syst Rev. 2016 Dec 21;12(12):CD010760. doi: 10.1002/14651858.CD010760.pub2. PMID: 28000926; PMCID: PMC6463967.
39. Plavoukou T, Staktopoulos P, Papagiannis G, Stasinopoulos D, Georgoudis G. Virtual and Augmented Reality for Chronic Musculoskeletal Rehabilitation: A Systematic Review and Exploratory Meta-Analysis. Bioengineering (Basel). 2025 Jul 8;12(7):745. doi: 10.3390/bioengineering12070745. PMID: 40722437; PMCID: PMC12292062.
40. Zheng L, Li X, Xu Y, Yang Y, Wan X, Ma X, Yao G, Li G. Effects of Virtual Reality-Based Activities of Daily Living Rehabilitation Training in Older Adults with Cognitive Frailty and Activities of Daily Living Impairments: A Randomized Controlled Trial. J Am Med Dir Assoc. 2025 Feb;26(2):105397. doi: 10.1016/j.jamda.2024.105397. Epub 2024 Nov 28. PMID: 39615543.
41. Saragih ID, Chen YM, Suarilah I, Susanto H, Lee BO. Virtual Reality Intervention for Fall Prevention in Older Adults: A Meta-Analysis. J Nurs Scholarsh. 2025 Sep;57(5):759-775. doi: 10.1111/jnu.70028. Epub 2025 Jun 18. PMID: 40533914.
42. Melillo A, Chirico A, De Pietro G, Gallo L, Caggianese G, Barone D, De Laurentiis M, Giordano A. Virtual Reality Rehabilitation Systems for Cancer Survivors: A Narrative Review of the Literature. Cancers (Basel). 2022 Jun 28;14(13):3163. doi: 10.3390/cancers14133163. PMID: 35804933; PMCID: PMC9264833.
43. Alhusamiah B, Aldiqs M, Zeilani RS. The Effectiveness of Immersive Virtual Reality as A Complementary Approach and a New Direction in Cancer Related Fatigue Management. Integr Cancer Ther. 2024 Jan-Dec; 23:15347354241280272. doi: 10.1177/15347354241280272. PMID: 39305200; PMCID: PMC11421400.
44. Toni E, Ayatollahi H. Enhancing cancer-supportive care through virtual reality: a policy brief. Health Res Policy Syst. 2025 Apr 29;23(1):52. doi: 10.1186/s12961-025-01332-w. PMID: 40301919; PMCID: PMC12039136.
